# Clinical significance of E-Cadherin and β-catenin in early gastric cancer: A protocol of systematic review

**DOI:** 10.1097/MD.0000000000022271

**Published:** 2020-10-09

**Authors:** Shu-fen Zhang, Jian-hua Zhang

**Affiliations:** a Department of Gastroenterology, Xi’an Gaoxin Hospital; b Department of Surgery, Xi’an Chest Hospital, Xi’an, Shaanxi, China.

**Keywords:** early gastric cancer, E-Cadherin, β-catenin

## Abstract

**Background:**

This study will summarize the clinical significance of E-Cadherin and β-catenin in early gastric cancer (EGC).

**Methods:**

Eligible case-control studies were searched from Cochrane Library, PUBMED, EMBASE, PsycINFO, Google Scholar, CBM, and CNKI from inception to the present. In addition, we will also search other sources to avoid missing potential studies. Two authors will independently carry out study selection, data collection, and study methodological quality. A fixed or random-effects model will be utilize to synthesize the data, and RevMan 5.3 software will be used for data analysis.

**Results:**

This study will summarize all eligible studies to investigate the clinical significance of E-Cadherin and β-catenin in EGC.

**Conclusion:**

The findings of this study may present a genuine understanding of perspective on the clinical significance of E-Cadherin and β-catenin in EGC.

## 1 Introduction

Gastric cancer is the second most common malignant tumors around the world.^[1–3]^ It is also the leading cause of cancer-related deaths worldwide.^[4,5]^ It has been reported that its 5-year survival rate is about 10% to 19% in the developed countries.^[6]^ In China, it is the most frequent diagnosed malignant tumors every year, with about 25% cancer-associated deaths.^[7]^ Thus, it is very important to diagnose at early stage, also known as early gastric cancer (EGC). Studies investigated the expression of E-Cadherin and β-catenin in EGC tissue.^[8–13]^ Several studies report the association between E-Cadherin, β-catenin and EGC; and both of them have clinical significance in EGC.^[14–22]^ However, all results are reported by individual study. No literature study systematically and comprehensively explores this topic. Therefore, this systematic review will investigate the clinical significance of E-Cadherin and β-catenin in EGC, which may provide helpful evidence for clinical practice.

## 2 Methods and analysis

### 2.1 Study registration

The present protocol has been registered on INPLASY202080072, and it has been reported based on the Preferred Reporting Items for Systematic Reviews and Meta-Analysis (PRISRMA) Protocol statement.^[23]^

### 2.2 Information sources and search strategy

This study will comprehensively retrieve all potential studies in both electronic databases and other literature sources. Electronic databases will be searched from inception to the present in Cochrane Library, PUBMED, EMBASE, PsycINFO, Google Scholar, CBM, and CNKI. The specifics of search strategy for PUBMED will be summarized in Table 1. We will modify similar search strategy to other electronic databases. In addition, we will examine other sources, such as thesis/dissertation, and conference abstracts to avoid missing potential studies.

**Table 1:**
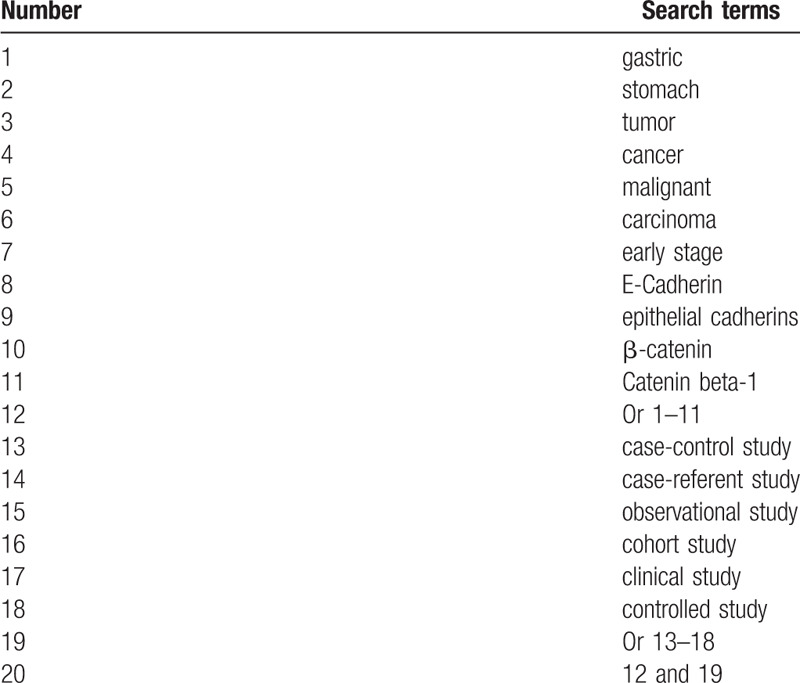
Search strategy for PUBMED.

### 2.3 Study eligibility criteria

#### 2.3.1 Participants

Patients with pathologically confirmed as EGC will be included without restrictions to sex, age, racial, and educational background.

#### 2.3.2 Intervention exposure

E-Cadherin and β-catenin in cancer tissue of patients with EGC were detected.

#### 2.3.3 Control exposure

E-Cadherin and β-catenin in adjacent normal tissue of patients with EGC were examined.

#### 2.3.4 Study designs

All eligible case-control studies (CCSs) on investigating the E-Cadherin and β-catenin in both cancer tissue and adjacent normal tissue of patients with EGC will be considered for inclusion, irrespective language and publication status.

#### 2.3.5 Outcomes

Primary outcomes are protein expression of E-Cadherin and β-catenin in both cancer and adjacent normal tissue. Secondary outcomes are gene expression of E-Cadherin and β-catenin in both cancer and adjacent normal tissue.

#### 2.3.6 Exclusion criteria

It includes insufficient essential data, animal study, review, comment, editorial, case report, case series, uncontrolled study, and other irrelevant studies.

### 2.4 Data collection and analysis

#### 2.4.1 Study selection

Endnote X7 software will be employed to manage all searched records, and all duplications will be removed. Then, 2 authors will check titles/ abstracts to eliminate irreverent citations. After that, the full text of all potential articles will be cautiously read to judge whether they fulfill all inclusion criteria. Divergences between both of them will be settled down by consulting a third experienced author. A flow diagram will be presented to describe study selection process of all searched records.

#### 2.4.2 Data collection and management

Two authors will be in charge of data collection independently based on the previously designed data collection form. Any confusion will be cleared up by a third author through discussion. The following data will be collected from included studies, including study characteristics (country, first author, time of publication, sample size, follow-up information, et al), patient characteristics (tumor stage, age, sex, ethnicity, pathological diagnosis, eligibility criteria, et al), study methods, study setting, information of intervention and comparator, outcomes (gene and protein of E-Cadherin and β-catenin), and other essential information.

#### 2.4.3 Missing data dealing with

Any missing data will be obtained from primary authors using email or phone. We will analyze available data only if we can not request those data.

### 2.5 Study quality assessment

Two authors will independently assess the study quality of all included CCSs using Newcastle-Ottawa scale.^[24]^ Any incompatibility difference will be solved by a third author through discussion.

### 2.6 Statistical analysis

We will use RevMan 5.3 software for statistical analysis. Continuous outcomes will be estimated as weighted mean difference (MD) or standard MD and 95% confidence intervals (CIs), and dichotomous outcomes will be estimated as risk ratio and 95% CIs. Heterogeneity across eligible CCSs will be checked using *I*^2^ test. *I*^2^ ≤ 50% will be considered as having reasonable heterogeneity, while *I*^2^ > 50% will be regarded as having substantial heterogeneity. A fixed-effects model will be employed to pool the data when statistical heterogeneity is absent. In addition, we will perform meta-analysis when sufficient data on the same outcome is collected. A random-effects model will be suggested to synthesize the data when statistical heterogeneity is significant. When necessary, we will carry out subgroup analysis and meta-regression analysis to explore possible sources of significant heterogeneity.

### 2.7 Additional analysis

We will conduct subgroup analysis and meta-regression analysis based on different study information, patient characteristics, and tumor stages. We will carry out sensitivity analysis to check robustness of study results by excluding low quality study. In addition, if more than 10 eligible CCSs on the same outcome are included, we will plan to perform funnel plot and Eggers regression test to investigate potential reporting bias.^[25,26]^

### 2.8 Ethics and dissemination

This study does not require ethical approval, because it will not collect individual patient data. We expect to publish this study on a peer-reviewed journal.

## 3 Discussion

Studies suggested that E-Cadherin and β-catenin were identified in EGC tissue.^[8–13]^ Other studies found that E-Cadherin and β-catenin are associated with EGC, and both of them have clinical significance in EGC.^[14–22]^ However, we do not identify insufficient evidence-based medicine evidence regarding this issue. With an increasing number of relevant studies, this proposed systematic review aims to investigate the clinical significance of E-Cadherin and β-catenin in EGC tissue. It will summarize most recent studies and synthesize data from them. Its results may provide helpful evidence for clinical practice and further studies.

## Author contributions

**Conceptualization:** Shu-fen Zhang, Jian-hua Zhang.

**Data curation:** Shu-fen Zhang, Jian-hua Zhang.

**Formal analysis:** Shu-fen Zhang, Jian-hua Zhang.

**Investigation:** Jian-hua Zhang.

**Methodology:** Shu-fen Zhang.

**Project administration:** Jian-hua Zhang.

**Resources:** Shu-fen Zhang.

**Supervision:** Jian-hua Zhang.

**Software:** Shu-fen Zhang.

**Validation:** Shu-fen Zhang, Jian-hua Zhang.

**Visualization:** Shu-fen Zhang, Jian-hua Zhang.

**Writing – original draft:** Shu-fen Zhang, Jian-hua Zhang.

**Writing – review & editing:** Shu-fen Zhang, Jian-hua Zhang.

## Abbreviations

Abbreviations: CCSs = case-control studies, CIs = confidence intervals, EGC = early gastric cancer, MD = mean difference, PRISRMA = Preferred Reporting Items for Systematic Reviews and Meta-Analysis.
